# Renal cell carcinoma with central nervous system demyelination caused by nivolumab

**DOI:** 10.1002/iju5.12234

**Published:** 2020-10-28

**Authors:** Toshiki Oka, Yoshiyuki Yamamoto, Yohei Okuda, Toshihisa Asakura, Koji Hatano, Yasutomo Nakai, Masashi Nakayama, Ken‐ichi Kakimoto, Fuminobu Sugai, Kazuo Nishimura

**Affiliations:** ^1^ Department of Urology Osaka International Cancer Institute Osaka Japan; ^2^ Department of Neurology Otemae Hospital Osaka Japan

**Keywords:** demyelination, immune checkpoint inhibitor, immune‐related adverse event, nivolumab, renal cell carcinoma

## Abstract

**Introduction:**

Central nervous system demyelination caused by immune checkpoint inhibitors is a very rare condition.

**Case presentation:**

A 65‐year‐old man who received nivolumab for renal cell carcinoma developed abnormal behavior, such as disagreeable speech and sudden anger. Brain‐enhanced magnetic resonance imaging revealed multiple lesions with partial contrast effects in the cerebral white matter. We tentatively diagnosed demyelination caused by nivolumab, and performed steroid pulse therapy twice. After that, his symptoms improved. For the next 2 years, his symptoms did not recur, nor did his cancer progress.

**Conclusion:**

Demyelination caused by immune checkpoint inhibitors can be fatal and requires early diagnosis and treatment.

**Abbreviations & Acronyms**
CNS: central nervous system
CT: computed tomography
CTCAE: Common Terminology Criteria for Adverse Events
ICI: immune checkpoint inhibitor
irAE: immune‐related adverse event
IVIG: intravenous immunoglobulin
MRI: magnetic resonance imaging
MS: multiple sclerosis
mPSL: methylprednisolone
PD‐1: programmed cell death 1
RCC: renal cell carcinoma

**Keynote message**

Although CNS demyelination owing to ICIs is a rare disorder, it causes neurological symptoms and can be fatal. Early diagnosis and treatment are crucial. Steroids may be required, depending on symptoms.

## Introduction

Nivolumab, an anti‐PD‐1 inhibitor, is widely used to treat various cancers, including RCC; however, numerous irAEs have been reported. Neurologic irAEs are relatively rare,^1^ especially neurologic irAEs with CNS demyelination. Here, we report an extremely rare case of RCC with CNS demyelination caused by nivolumab.

## Case presentation

**Fig. 1:**
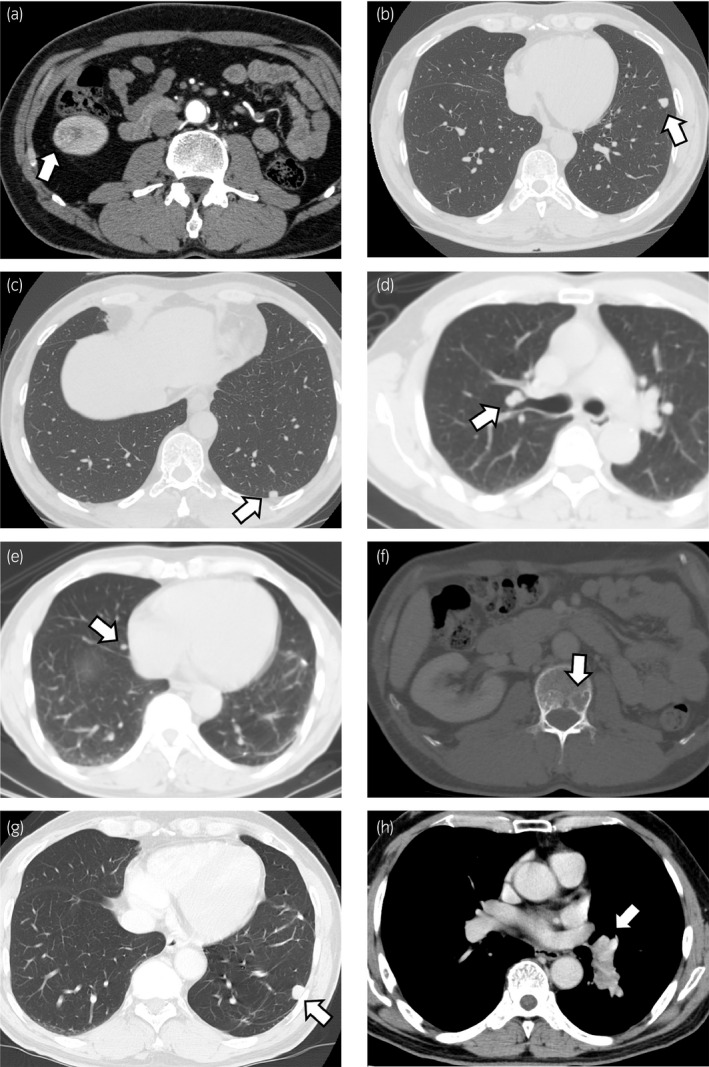
The CT images are shown. An arrow indicates a metastatic lesion. (a) 16‐mm enhanced mass in the lower pole of right kidney; (b) 9‐mm coin lesion in the upper lobe of left lung; (c) 8‐mm coin lesion in the lower lobe of left lung; (d) 8‐mm coin lesion near the hilum of right lung; (e) 5‐mm coin lesion in the middle lobe of right lung; (f) 15‐mm osteolytic lesion in the second lumbar spine; (g) 13‐mm coin lesion in the lower lobe of left lung; (h) 42‐mm left hilar lymph node.

A 52‐year‐old man underwent a left nephrectomy in another hospital for left RCC in 2004 (pathological diagnosis unknown). He developed left lung metastases in 2010, and started treatment with interferon‐α. Right renal metastasis also appeared in 2010 (Fig. 1a), so he was referred to our hospital and underwent a right partial nephrectomy. Later, he also underwent a left partial pneumonectomy (Fig. 1b,c). The histopathological finding of each excised tissue showed clear cell RCC. In April 2015, he began sunitinib treatment for multiple lung metastases (Fig. 1d,e) (International Metastatic RCC Database Consortium risk group was favorable), but a lumbar spine metastasis was found in February 2016 (Fig. 1f). His medication was switched from sunitinib to axitinib in November 2016. In October 2017, he began taking nivolumab because of the progression of lung metastases and appearance of left hilar lymph node disease (Fig. 1g,h). In January 2018, he received a transarterial embolization for his left hilar lymph node, because of progressive disease. Both the hilar lymph node and lung disease showed durable responses.

**Fig. 2:**
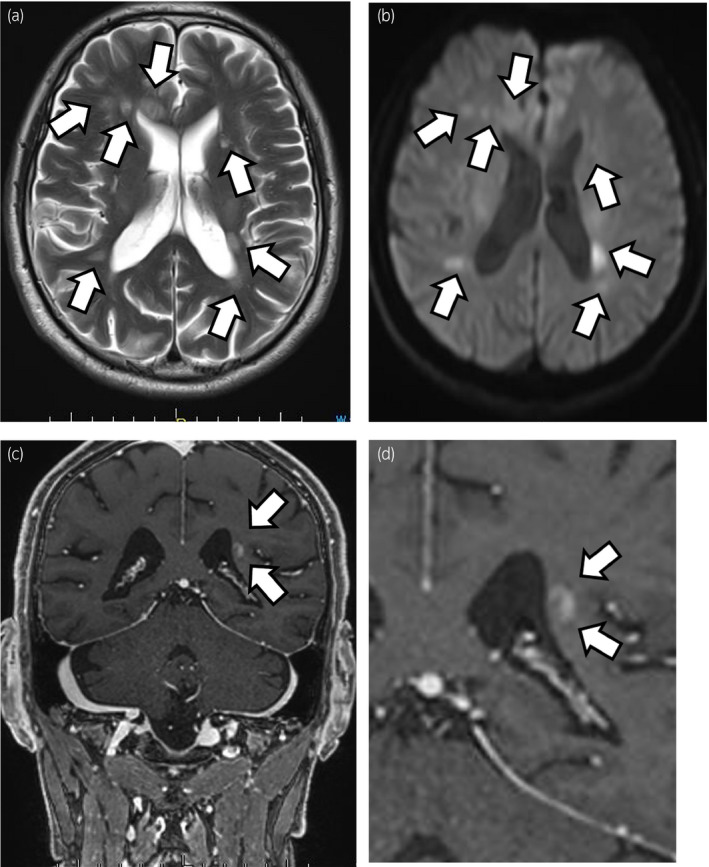
Brain MRI shows high signal in T2‐weighted images and diffusion‐weighted images in the cerebral white matter (arrows: CNS demyelination). (a) T2‐weighted images; (b) diffusion‐weighted images; (c) brain‐enhanced MRI shows an open‐ring sign; (d) enlarged image of panel c.

Three days after his 11th nivolumab administration, he began displaying abnormal behavior, such as disagreeable speech and sudden anger. Eleven days later, he also developed a short‐term memory loss and calculation disorder and was hospitalized on the same day. Brain MRI showed multiple lesions, with high signals in T2‐weighted images in his cerebral white matter (Fig. 2a,b). Their open‐ring signs suggested demyelination rather than metastatic tumors (Fig. 2c,d). Demyelination caused by nivolumab was considered to be likely, although we need to rule out infectious diseases, collagen diseases, and MS. His cerebrospinal fluid showed normal glucose, protein, and white blood cell count, presence of oligoclonal bands; normal levels of myelin basic protein, immunoglobulin G, and immunoglobulin A for toxoplasma, and negative JC viral DNA. No malignant cells were found in the cerebrospinal fluid. Most autoantibodies, including anti‐aquaporin 4 antibody, were negative except anti‐nuclear antibody. On the basis of the above examinations, we diagnosed CNS demyelination caused by nivolumab, which was classified as a grade 2 adverse effect in accordance with the CTCAE version 5.0.

**Fig. 3:**
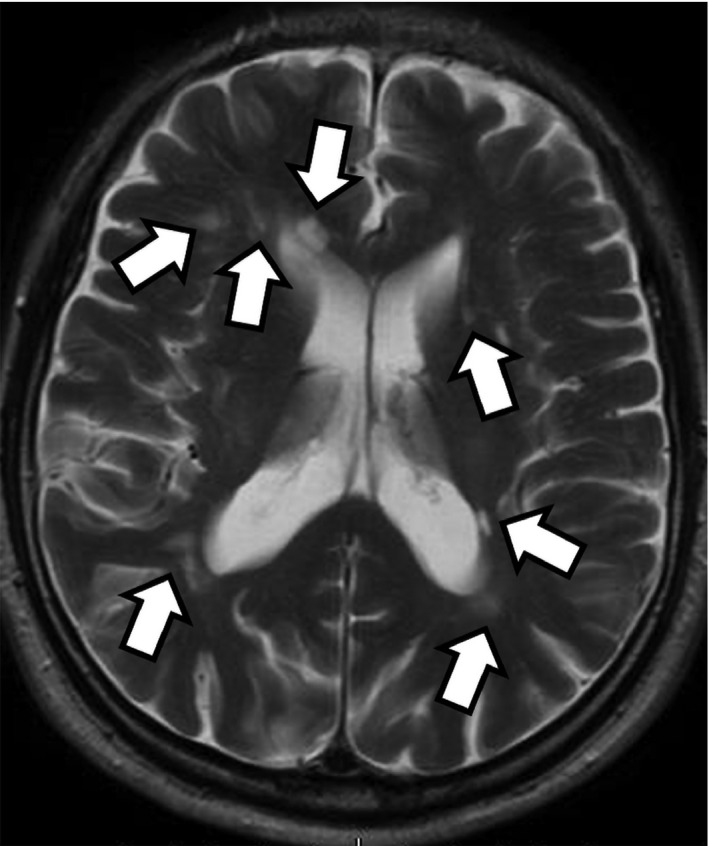
Brain MRI (T2‐weighted images) taken 6 months after discharge showed further improvement of multiple lesions in the cerebral white matter (arrows: CNS demyelination).

Nivolumab was ceased and intravenous mPSL (1 g/day) was administered for 3 days from the day of his hospital admission. However, as his neurological symptoms did not greatly improve, we began intravenous mPSL (1 g/day) again for 3 days from the eighth day of hospitalization. He then began to show improvement of abnormal behavior as well as imaging findings. Neurological symptoms, such as disagreeable speech and sudden anger, subsided completely. He was discharged on the 23rd hospital day and fully recovered a short‐term memory loss and calculation disorder 3 months after the onset. No steroid was administered other than intravenous mPSL for a total of 6 days. After 6 months, his brain MRI showed further improvement of multiple lesions in the cerebral white matter (Fig. 3). Nivolumab has been discontinued and neither neurologic symptoms nor progression of RCC have been observed for 26 months without any treatment.

## Discussion

A report of 9208 patients treated with ICIs, enrolled in 59 clinical trials, showed that 3.8% of patients were treated with cytotoxic T‐lymphocyte‐associated protein‐4 inhibitor, 6.1% of those with PD‐1 inhibitor and 12.0% of those with both agents had neurologic irAEs.^1^ Yet, neurologic irAEs with CNS demyelination are rare conditions. Neurologic irAEs encompass a broad spectrum of neurologic syndromes, including myasthenic syndrome, aseptic meningitis, encephalitis, sensory motor neuropathy, Guillain‐Barré‐like syndromes, painful sensory neuropathy, enteric neuropathy, transverse myelitis, and posterior reversible encephalopathy syndrome.^2^ To diagnose neurologic irAEs, infectious diseases, cancer invasion to CNS, paraneoplastic syndrome, and metabolic disorders must be ruled out. Blood tests that include hormone levels, cerebrospinal fluid tests, and brain‐enhanced MRI are essential.^3^ In patients with CTCAE grade 1 neurologic symptoms, ICIs should be continued under close observation. Among those with CTCAE grade ≥2 neurological symptoms, administration of corticosteroids equivalent to mPSL (1–4 mg/kg), and discontinuation of ICIs, or treatment such as IVIG, plasma exchange, and steroid pulse are required depending on severity.^2^

To our knowledge, only eight cases of CNS demyelination owing to ICIs have been reported, including the present case (Table 1).^4^, ^5^, ^6^, ^7^, ^8^, ^9^, ^10^ Our report is the first case of CNS demyelination caused by nivolumab to treat RCC. All other reported patients were treated with steroids except for one patient with no symptoms who was relieved by only discontinuation of nivolumab. IVIG, plasma exchange, and immunosuppressant were added in some cases. In previous reports, most patients with irAEs involving the CNS had onset of symptoms <4 days after administering nivolumab, and symptoms became progressively worse within 2 weeks.^8^ Except for one patient with unknown details, all eight patients improved in response to treatment. However, one died from relapsed CNS demyelination, and two died from complications of steroid treatment. As four cases out of eight died, CNS demyelination caused by ICIs can be fatal. We tried the second steroid pulse therapy in the same manner as for MS, which is a CNS demyelinating disease with a clinical picture similar to our case. Since the neurological symptoms were not severe and subsided after the second administration of mPSL, we decided to follow‐up carefully without continuing steroid administration.

**Table 1:** Review of cases of cerebral nervous system demyelination secondary to treatment with ICIs

Case	Author/year	Underlying disease	Suspected drug	Symptom	Treatment provided	Responsiveness to treatment	Outcome
1	Maurice *et al.* ^4^/2015	Melanoma	Nivolumab	Confusion	mPSL	After remission, symptoms reappear	Died due to demyelinating disease
				Nausea	IVIG		
				Vomiting			
				Apathy			
				Fixed gaze			
				Psychomotor slowing			
2	Cao *et al*.^5^/2016	Melanoma	Ipilimumab	Fatigue	mPSL	Unknown	Died
				Memory loss	Cyclophosphamide		
				Vision change			
3	Sugiura *et al*.^6^/2017	Lung cancer	Nivolumab	Low motivation	mPSL	Improvement	Died due to cytomegalovirus ulcer in the lower intestinal tract
				Wobbling			
4	Mancone *et al*.^7^/2018	Melanoma	Nivolumab	Gait imbalance	mPSL	Mild improvement	Died due to urinary tract infection
			Ipilimumab	Lower extremity weakness	IVIG		
5	Zafer *et al*.^8^/2019	Laryngeal cancer	Nivolumab	Diffuse generalized slowing	mPSL	Improvement	Survived
					IVIG		
6	Pillonel *et al*.^9^/2019	Melanoma	Nivolumab	No symptom	Only discontinue of nivolumab	Improvement	Survived
7	Duraes *et al*.^10^/2019	Melanoma	Pembrolizumab	Distal numbness of the limbs	Intravenous steroid	Improvement	Survived
				Weakness with gait impairment	Plasma exchange		
8	Our case	Renal cell	Nivolumab	Disagreeable speech Sudden anger Memory loss Calculation disorder	mPSL	Improvement	Survived
		carcinoma					

An autopsy for a patient with CNS demyelination revealed infiltration of CD8^+^ T cells into the margin of the demyelinating lesion.^4^ Furthermore, a pathway in which CD8^+^ T cells damaged the myelin sheath has been reported for MS.^11^ Furthermore, others have reported that the autopsy of MS patients showed no PD‐1 expression by CD8^+^ T cells that infiltrate the lesion site.^12^ These facts support the hypothesis that the CD8^+^ T cells whose PD‐1 receptors are inhibited by nivolumab may injure the myelin sheath and cause CNS demyelination.

## Conclusion

We experienced a case of RCC with demyelination of the CNS owing to nivolumab. Because this condition may be fatal, early diagnosis and treatment are necessary.

## Conflict of interest

The authors declare no conflict of interest.
